# Impacts of human pressure and climate on biodiversity–multifunctionality relationships on the Qinghai–Tibetan Plateau

**DOI:** 10.3389/fpls.2023.1106035

**Published:** 2023-05-30

**Authors:** Chongchong Ye, Shuai Wang, Yi Wang, Tiancai Zhou, Ruowei Li

**Affiliations:** ^1^ State Key Laboratory of Earth Surface Processes and Resource Ecology, Faculty of Geographical Science, Beijing Normal University, Beijing, China; ^2^ School of Life Sciences and State Key Lab of Biological Control, Sun Yat-sen University, Guangzhou, China; ^3^ Synthesis Research Centre of Chinese Ecosystem Research Network, Key Laboratory of Ecosystem Network Observation and Modelling, Institute of Geographic Sciences and Natural Resources Research, Chinese Academy of Sciences, Beijing, China; ^4^ College of Grassland, Resource and Environment, Inner Mongolia Agricultural University, Hohhot, China

**Keywords:** ecosystem multifunctionality, human pressure, aridity, Qinghai–Tibetan Plateau, biodiversity–multifunctionality relationships

## Abstract

Many studies have investigated the effects of environmental context on biodiversity or multifunctionality in alpine regions, but it is uncertain how human pressure and climate may affect their relationships. Here, we combined the comparative map profile method with multivariate datasets to assess the spatial pattern of ecosystem multifunctionality and further identify the effects of human pressure and climate on the spatial distribution of biodiversity–multifunctionality relationships in alpine ecosystems of the Qinghai–Tibetan Plateau (QTP). Our results indicate that at least 93% of the areas in the study region show a positive correlation between biodiversity and ecosystem multifunctionality across the QTP. Biodiversity–multifunctionality relationships with increasing human pressure show a decreasing trend in the forest, alpine meadow, and alpine steppe ecosystems, while an opposite pattern was found in the alpine desert steppe ecosystem. More importantly, aridity significantly strengthened the synergistic relationship between biodiversity and ecosystem multifunctionality in forest and alpine meadow ecosystems. Taken together, our results provide insights into the importance of protecting and maintaining biodiversity and ecosystem multifunctionality in response to climate change and human pressure in the alpine region.

## 1 Introduction

One of the primary directions for ecology is to determine how biodiversity is generated and maintained across broad macroecological patterns and temporal dynamics (Chase, 2010; Guo et al., 2019), which can enhance our overall awareness of the underlying mechanisms shaping biodiversity, the development of ecological theories, and the conservation of biodiversity (Zhou et al., 2008; Morlon et al., 2011; Guo et al., 2019). Most studies have demonstrated that biodiversity and ecosystem functions are closely related (Jing et al., 2015; Gross et al., 2017; Hu et al., 2021). More importantly, their relationships can further influence ecosystem services related to human well-being (Cameron et al., 2018), including the quantity and quality of the food we eat, the water we drink, and the air we breathe and the control of disease (Wall et al., 2015). However, with increasing human pressures and climate change (Yao et al., 2022), the relationships between biodiversity and multifunctionality may be subject to great uncertainty in response to these changes. There is therefore an urgent need to improve comprehensive information on biodiversity–multifunctionality relationships at spatially explicit scales in the context of global change.

At present, field experiments (Delgado-Baquerizo et al., 2020), belt transect surveys (Jing et al., 2015; Hu et al., 2021), and meta-analyses (Zhang R. et al., 2022) have shown that biodiversity plays an important role in driving ecosystem functioning at different trophic levels. The multiple lines of evidence suggest that the relationships between biodiversity and ecosystem functioning can be strongly predicted by the biodiversity of plant and soil microbiota (Maestre et al., 2012; Meyer et al., 2018; Albrecht et al., 2021) and that there is the potential for the synergistic and complementary influence of plant (Fanin et al., 2018; Valencia et al., 2018) and soil microbial diversity (Zhang and Zhang, 2016; Delgado-Baquerizo et al., 2020) on ecosystem functioning. For example, an extensive climatic gradient study on the Qinghai–Tibetan Plateau (QTP) showed that the observed differences in ecosystem multifunctionality between sites were largely (45%) due to the joint influence of above- and belowground biodiversity (Jing et al., 2015). As such, the aridity gradient study in northern China showed that soil multifunctionality has a strong positive association with plant species richness in less arid regions and with microbial diversity in more arid regions, respectively (Hu et al., 2021). Yet, in the context of human-induced global climate change, biodiversity and ecosystem multifunctionality may be subject to potential ecological consequences under uncertain environmental change (Wagg et al., 2014; Jing et al., 2015; Delgado-Baquerizo et al., 2020; Hu et al., 2021). For instance, human-induced land-use changes, such as desertification, urbanization, deforestation, and agriculture, can cause profound destruction of biodiversity and ecosystem functions (Haddad et al., 2015; Tsiafouli et al., 2015; Wall et al., 2015). Similarly, increased soil erosion caused by extreme water (Wall et al., 2015) and wind events (Garrison et al., 2003) can alter the intrinsic structure of dust storms and the distribution of soil organisms and pathogens (Quinton et al., 2010), with significant impacts on the decomposition function of ecosystems. Although these works provide multidimensional perspectives to demonstrate the importance of environmental context in maintaining biodiversity and ecosystem multifunctionality, there is still a lack of macro-ecological perspectives and determinations of the impacts of human pressure and climate on their relationships across specific ecological gradients, such as the QTP.

The QTP region is one of the world’s biodiversity hotspots and contains numerous endemic amphibians, mammals, birds, and plants, especially along the eastern Himalayas and the Hengduan Mountains (Fu and Shen, 2017; Deng et al., 2020; Yu et al., 2021). A previous report indicated that the Hengduan Mountains are home to 8,439 species of seed plants and that a total of 358 species of passerine birds can be found along the gradient of forest ecosystems that appears to be warm and humid in the eastern Himalayas (Päckert et al., 2020; Yu et al., 2020; Mao et al., 2021). These unique ecological conditions combined with the environmental background have made the QTP a “natural experimental site,” triggering a lot of scientific research, mainly on plant phenology (Shen et al., 2022), cryospheric environment transformation (Kang et al., 2010), water supply changes (Immerzeel et al., 2010), alpine forest growth (Silva et al., 2016), alpine ecosystem vegetation shifts (Zhang et al., 2013), ecosystem function (Zhang H. et al., 2022), and ecosystem services (Jiang et al., 2020). In comparison, although there is important research on the effects of climatic variation and biodiversity on ecosystem functions at the plot level (Jing et al., 2015; Wang Y. et al., 2021), there is a lack of studies describing the spatial distribution of ecosystem multifunctionality and biodiversity–multifunctionality relationships and how this relationship responds to human pressure and climate change along different ecological gradients.

Here, we used multivariate data (including total N, total P, total K, alkali-hydrolyzable N, available P, available K, cation exchange capacity, soil organic matter, carbon stock density of above- and belowground living biomass, soil microbial biomass carbon and nitrogen, vascular plant species richness, and hydrothermal conditions) based on the averaging approach, principal component analysis (PCA) approach, threshold approach, and Comparison Map Profile Method to assess the impacts of human pressure and climate on the spatial distribution of biodiversity–multifunctionality relationships across different ecosystems of the QTP. Our main objectives were to (1) assess the spatial distribution of ecosystem multifunctionality based on averaging, PCA, and threshold approaches across the QTP and validate the robustness of the assessment results; (2) identify the spatial distribution of biodiversity–multifunctionality relationships across the QTP; (3) assess the impacts of human pressure and climate on the spatial distribution of biodiversity–multifunctionality relationships across different ecosystems of the QTP. We provide insights that can guide future conservation and maintenance of biodiversity and ecosystem multifunctionality in response to climate change and human pressure in the alpine region.

## 2 Materials and methods

### 2.1 Study area

**Figure 1:**
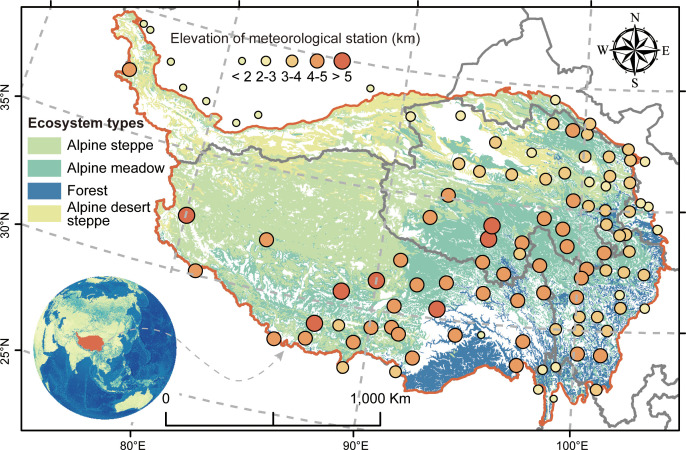
The distribution of ecosystem types used in this study. The illustration at the bottom left denotes the location of the Qinghai–Tibetan Plateau around the world. The points of different proportions represent the elevation changes of meteorological sites.

Being the largest area of ice and snow storage apart from the Arctic and Antarctic, the QTP possesses a mean elevation of over 4,000 m and an area of ~2.50 × 10^6^ km^2^ (Zhang et al., 1999; Wu and Zhang, 2010; Kou et al., 2020), with a representative dry and cold climate, mainly influenced by the Indian summer monsoon and East Asian monsoon. Because of the gradual decrease of the East Asian monsoon with the altitudinal gradient from southeast to northwest, the climate pattern correspondingly represents the identical gradient change, with ranges of mean annual precipitation from 1,000 to 100 mm and ranges of mean annual temperature from 7.4°C to -4.1°C (Kou et al., 2020; Sun et al., 2020). These geographic variations of climate and topographic conditions conjointly characterize the particular ecosystem type on the QTP, spanning forest, alpine meadow, alpine steppe, and alpine desert steppe from southeast to northwest (**Figure 1**). As the primary ecosystem throughout the QTP, the alpine steppe is chiefly dominated by *Stipa purpurea* and *Carex moorroftii*, whereas in the alpine meadow, *Kobresia pygmaea* and *Kobresia humilis* are the dominant species (Ding et al., 2017). In brief, these exchanges of matter, energy, and organisms across different ecosystems shape the particularity of QTP around the world (Scherer-Lorenzen et al., 2022).

### 2.2 Climate and human pressure datasets

We employed the interpolation of precipitation and temperature to evaluate the effect of climate on biodiversity–multifunctionality relationships across the QTP. The interpolation of climate data (mainly including monthly mean air temperature and monthly precipitation sum) was conducted on the Anusplin 4.2 software (Centre for Resource and Environmental Studies, Australian National University, Canberra) by using the 107 meteorological sites (**Figure 1**) that were obtained from the China Meteorological Administration (http://cdc.cma.gov.cn) with a 5-km spatial resolution for the period 1982–2010. To evaluate the effect of human pressure on biodiversity–multifunctionality relationships across the geographical gradient of the QTP, we employed the index of global human modification to reflect it, which was obtained from the Global Human Modification of Terrestrial Systems (https://sedac.ciesin.columbia.edu/data/set/lulc-human-modification-terrestrial-systems) with a 1-km resolution and contained specific classification criterion: none (value = 0), low (0< value ≤ 0.1), moderate (0.1< value ≤ 0.4), high (0.4< value ≤ 0.7), very high (0.7< value ≤ 1), and uncategorized (value = NA).

### 2.3 Ecosystem function and biodiversity datasets

To assess the ecosystem functions provided by different sources, we obtained total N, total P, total K, alkali-hydrolyzable N, available P, available K, cation exchange capacity, and soil organic matter products from the Land-Atmosphere Interaction Research Group at Sun Yat-Sen University (http://globalchange.bnu.edu.cn/research/soil2), with a 30-arcsec resolution in a 0–0.045-m-depth soil. Meanwhile, we also acquired the above- and belowground living biomass carbon stock density from ORNL DAAC (https://daac.ornl.gov/VEGETATION/guides/Global_Maps_C_Density_2010.html) at a spatial resolution of 300 m. The soil microbial biomass carbon and nitrogen are freely available from the Compilation of Global Soil Microbial Biomass Carbon, Nitrogen, and Phosphorus Data (http://daac.ornl.gov/cgi-bin/dsviewer.pl?ds_id=1264) with a spatial resolution of 0.5°. In this study, all of the ecosystem functions are employed to represent how the ecosystem is functioning in relation to relevant ecosystem services, chiefly providing a proxy of ecosystem function linked to soil fertility, nutrient cycling, potassium cycling, phosphorus cycling, carbon cycling, the composition of carbon pools, the dynamics of organic decomposition, and nitrogen mineralization (Xu et al., 2013; Soliveres et al., 2014). Furthermore, to comprehend the potential level of plant biodiversity across the QTP, we obtained a vascular plant species richness product (Kreft and Jetz, 2007) from the Laboratory for Anthropogenic Landscape Ecology (https://ecotope.org/anthromes/biodiversity/plants/maps/) at a spatial resolution of 1°. All grid products described above were resampled to 5 km spatial resolution to facilitate comparison.

### 2.4 Assessment of ecosystem multifunctionality based on different approaches

To detect the integrated ability of different forms of ecosystem functions across the QTP, we employed averaging, PCA, and threshold approaches to assess the ecosystem’s multifunctionality. The averaging approach collects the average values of each standardized ecosystem function in each grid; here, we calculated the standardized values using the Z-scores as described in Gross et al. (2017). For the PCA approach, we calculated the PCA axis at a grid-scale using the Principal Component Toolbox of ArcMap 10.7 (Environmental Systems Research Institute, Inc., Redlands, CA, USA), based on the standardized indicators of ecosystem functions. We then summed the PCA axis scores weighted by the contribution rate of each axis in groups into a single index to reflect the integrated ability of ecosystem functions (Meyer et al., 2018). In addition to the two approaches described above, we also conducted the threshold approach to generate the spatial maps of ecosystem multifunctionality. For the local-scale studies, the multiple-threshold approach can be readily realized by the most significant relationship between biodiversity and multifunctionality (that is, the capacity of biodiversity to sustain multiple ecosystem functions at high levels). In contrast, given that the ecosystem’s multifunctionality on a widespread scale, there is unprecedented heterogeneity of threshold in each grid spanning the QTP, which will lead to the calculation of multifunctionality inapplicably using the multiple-threshold approach based on the ecosystem functions without time series; thus, we utilized the recommendation as described by Gamfeldt et al. (2008). They employed a single threshold to quantify ecosystem multifunctionality and selected 50% of the maximum for each ecosystem function as the threshold, following the next two reasons: (1) If an ecosystem can provide 50% of the maximum species for a function, thereby considering that the ecosystem can maintain this function by referencing the median lethal effect concentration in ecotoxicology; and (2) For any single function, on average one species was needed to maintain a specific process at ~60% of the maximum rate. Thus, selecting 50% as the threshold can maximize the probability, i.e., one function at least needs a species to sustain. It is important to note that the maximum represents the range at the top 5% of each ecosystem function, and that is to prevent outliers. After determining the threshold and maximum, we counted the total number of ecosystem functions that passed the prescribed threshold and thereby produced the spatial map of ecosystem multifunctionality with the threshold approach. Although we used three basic approaches to assess ecosystem multifunctionality at the grid scale, each approach has its strengths and weaknesses. To address this issue, we average the three indexes after normalization to synthetically represent ecosystem multifunctionality.

### 2.5 Characterizing aridity level

We employed the aridity index to introduce the aridity extent across the QTP, which was the ratio between precipitation and potential evapotranspiration (i.e., aridity index = precipitation/potential evapotranspiration). To quantize the potential evapotranspiration at grid scale across the QTP, we utilized the Penman–Monteith method based on the interpolation data of monthly mean air temperature and monthly precipitation sum. The detailed calculation process is well explained in a previous study (Vicente-Serrano et al., 2010). Finally, we adopted the value of 1 − aridity index to reflect the aridity level, where higher index values represent drier conditions, and *vice versa* (Berdugo et al., 2017).

### 2.6 Assessment of biodiversity–multifunctionality relationships at grid scale

In this study, we used the Comparison Map Profile Method to examine biodiversity-multifunctionality relationships at each grid. The Comparison Map Profile Method employs the averaged values of cross-correlation (CC) coefficient from scales 1 (9 × 9 pixel moving window) to 10 (99 × 99-pixel moving window), increasing by 10 increments to represent biodiversity–multifunctionality correlations; their computational formula is as follows (Yao et al., 2018; Ding et al., 2019):


(1) $$\text{CC}=\frac{1}{N^{2}}\sum_{i=1}^{N}\sum_{j=1}^{N}\frac{\left(x_{ij}-\bar{x})(y_{ij}-\bar{y}\right)}{\sigma_{x}\sigma_{y}}$$

with


(2) $$\sigma_{x}^{2}=\frac{1}{N^{2}-1}\sum_{i=1}^{N}\sum_{j=1}^{N}{\left(x_{ij}-\bar{x}\right)}^{2}$$

where $\bar{x}$ and $\bar{y}$ denote the averaged values of two moving windows for the two comparative images of biodiversity and ecosystem multifunctionality. The *x_ij_* and *y_ij_* represent the pixel value of two moving windows in two compared images at row *i* and column *j*, respectively. *σ_x_* and *σ_y_* denote the standard deviation corresponding to the two moving windows of two comparative images. The *N* is the pixel number of moving windows. Usually, high CC values illustrate that the two comparative images possess better synergic relationships, and *vice versa*.

### 2.7 Analyses

We first assessed the spatial distribution of ecosystem multifunctionality across the QTP and used the biodiversity dataset to verify the robustness of the results. To detect the relationship between plant species richness and ecosystem multifunctionality at grid scale across the QTP, we applied the Comparison Map Profile Method to quantify the correlation between native species richness (NSR) and multifunctionality index (MI). After obtaining the biodiversity–multifunctionality relationships at the grid scale, we then adopted the “GAM” of smooth function in the R 4.1.1 software to examine the variations of biodiversity–multifunctionality relationships with human pressure in different ecosystems. We also analyzed the data distribution characteristics of biodiversity–multifunctionality relationships, from none to the high extent of human pressure in different ecosystems. Given that very high human pressure is principally distributed in urban land, we did not count the distribution of biodiversity–multifunctionality relationships in this type. Finally, we conducted the bivariate correlations between biodiversity–multifunctionality relationships and the multiyear average of precipitation, temperature, or aridity in different ecosystems by using the “lm” function in the R 4.1.1 software.

## 3 Result

### 3.1 Spatial distribution of ecosystem multifunctionality and biodiversity–multifunctionality relationships

**Figure 2:**
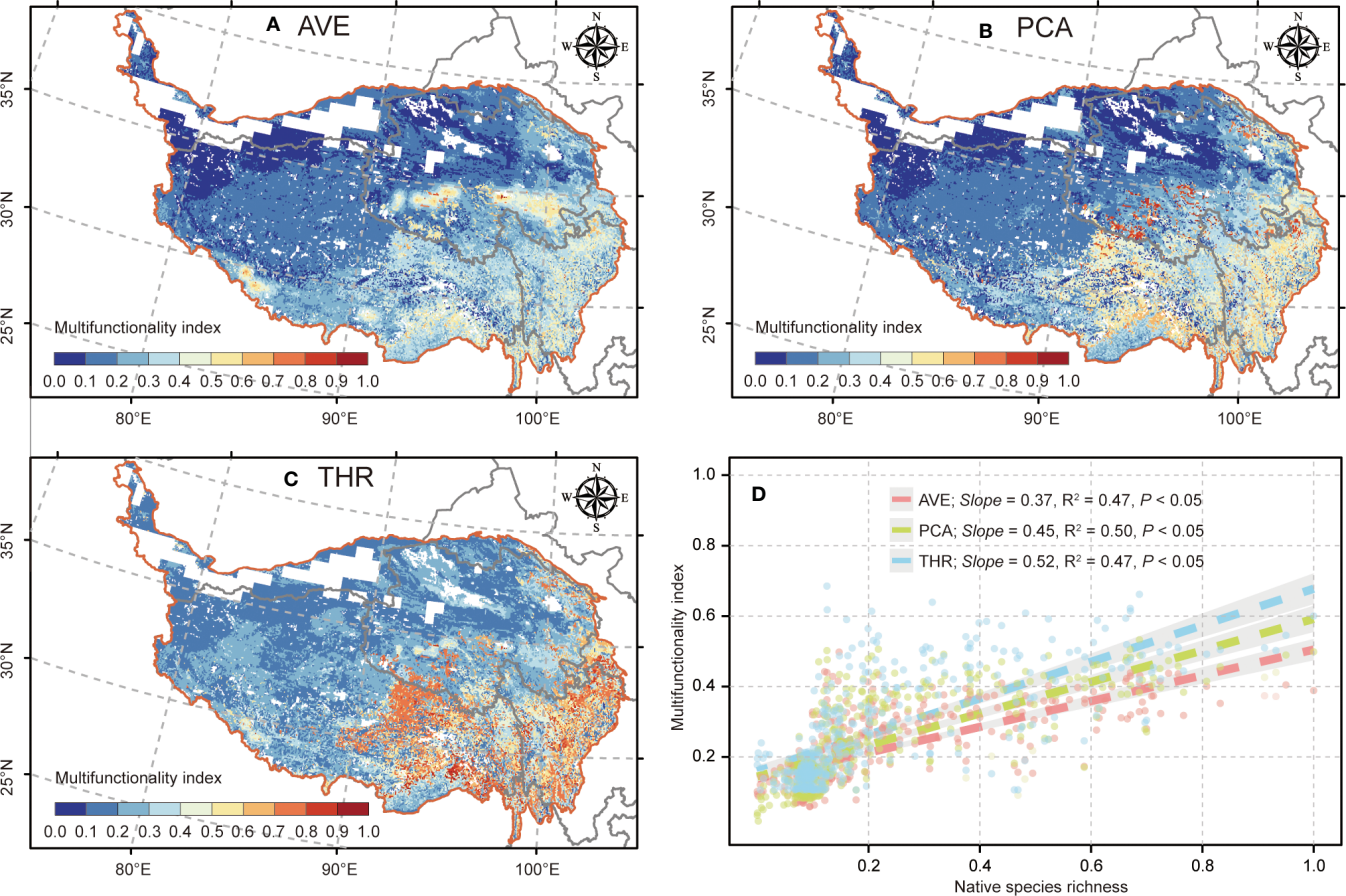
The spatial distribution of ecosystem multifunctionality based on different approaches and evaluation of robustness. **(A–C)** The spatial patterns of ecosystem multifunctionality calculated based on the averaging (AVE), principal component analysis (PCA), and threshold (THR) approaches on the Qinghai–Tibetan Plateau, respectively. **(D)** Bivariate correlations between ecosystem multifunctionality and native species richness of plants. Shading denotes the 95% confidence interval of the regression.

The spatial pattern of ecosystem multifunctionality shows that three indexes experienced relatively consistent geographic variations, i.e., with the high value primarily occurring in the southeast (≥ 0.60; **Figures 2A–C**) and the low value chiefly distributed in the northwest region (≤ 0.20; **Figures 2A–C**), whereas the ecosystem multifunctionality based on averaging approach owned fewer high-value areas when compared with PCA and threshold approaches in the southeast of QTP (**Figure 2A**). Additionally, the three ecosystem multifunctionality indexes displayed excellent robustness and experienced significant bivariate correlations (*R* ^2 ^= 0.49, 0.49, and 0.48 for averaging, PCA, and threshold approaches, respectively; *p *< 0.05; **Figure 2D**) with plant species richness.

**Figure 3:**
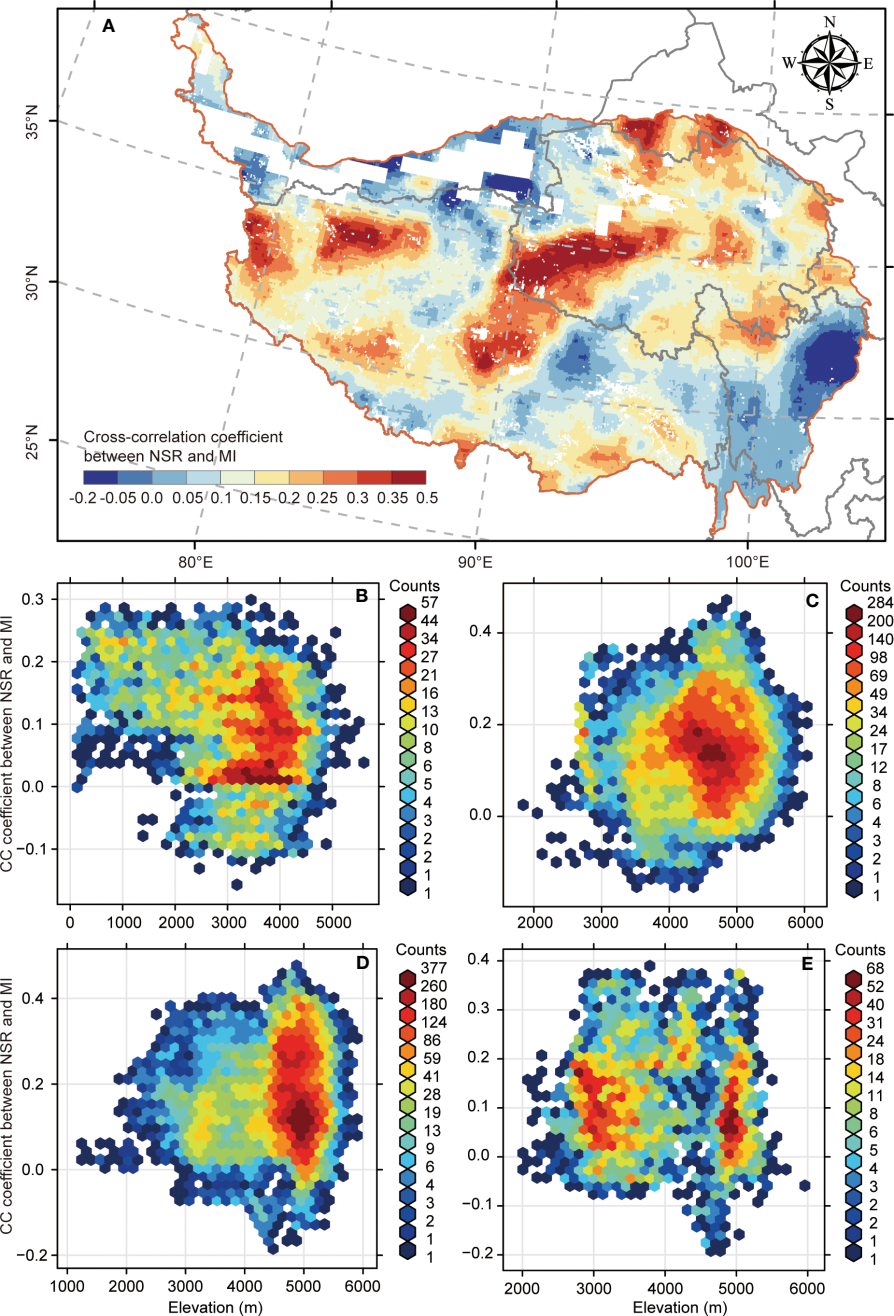
The spatial distribution of biodiversity–multifunctionality relationships and its variation with elevation. **(A)** The spatial distribution of biodiversity–multifunctionality relationships across the Qinghai–Tibetan Plateau. **(B–E)** Variations of biodiversity–multifunctionality relationships with elevation in the forest, alpine meadow, alpine steppe, and alpine desert steppe ecosystems, respectively.

For the biodiversity–multifunctionality correlations at the grid scale across the QTP, we found that more than 90% of the region (93.44%; **Figure 3A**) possessed a synergic relationship (CC > 0). Meanwhile, there were considerable heterogeneous biodiversity–multifunctionality correlations that possessed high values (CC exceeding or equal to 0.3) chiefly concentrating in the central region of QTP (**Figure 3A**) and low values (CC ≤ 0.05) primarily distributed in the eastern region of QTP (**Figure 3A**). For the variations of biodiversity–multifunctionality relationships with elevation, we found a decrease in biodiversity–multifunctionality correlation with elevation ascent in the forest (**Figure 3B**), yet the alpine meadow experienced the opposite tendency (**Figure 3C**). Additionally, the alpine steppe and alpine desert steppe underwent no clear change for biodiversity–multifunctionality correlation across altitudinal gradient (**Figures 3D, E**).

### 3.2 The effect of human pressure on biodiversity–multifunctionality relationships

**Figure 4:**
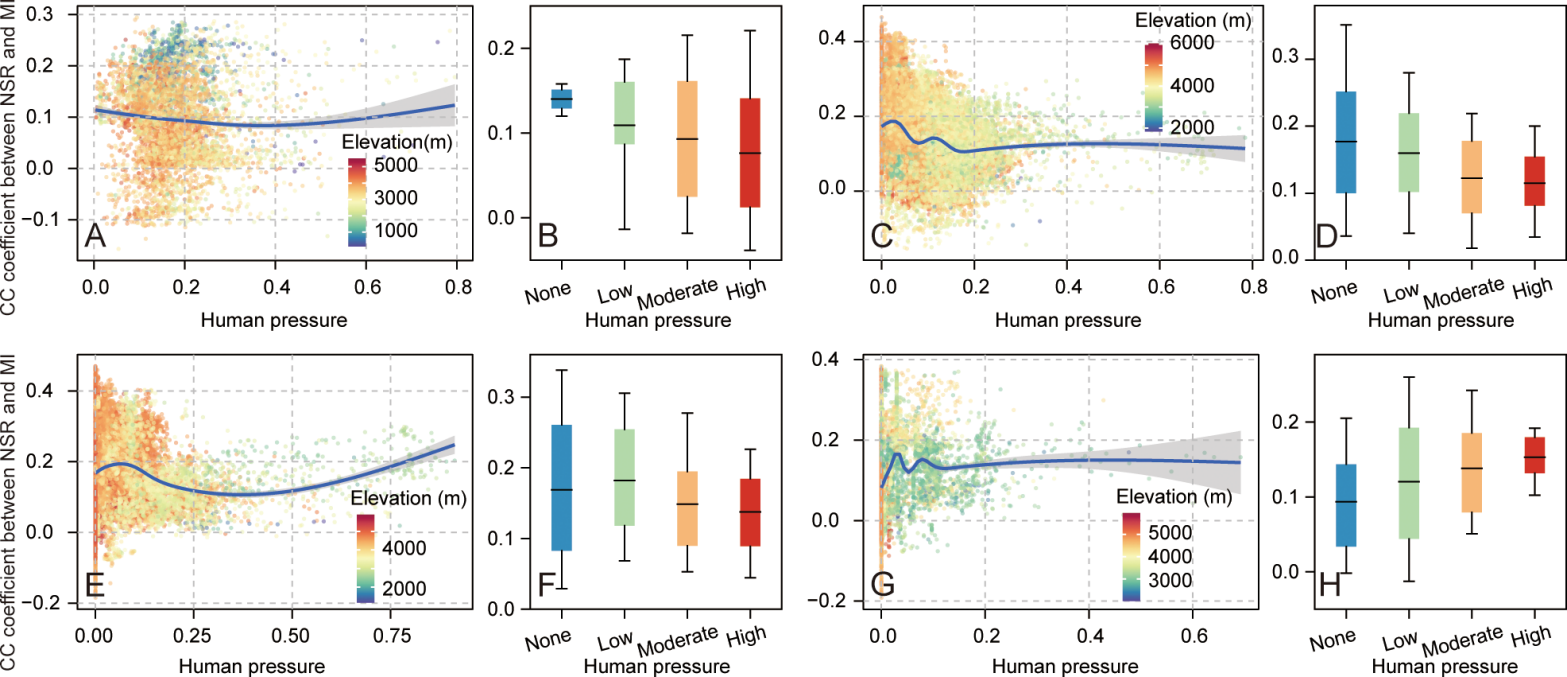
The human pressure in relation to biodiversity–multifunctionality correlations in different ecosystems. **(A**, **C**, **E**, **G)** Variations of biodiversity–multifunctionality relationships with increased human pressure in the forest, alpine meadow, alpine steppe, and alpine desert steppe ecosystems, respectively. **(B**, **D**, **F**, **H)** The statistics of biodiversity–multifunctionality relationships in different extents of human pressure for the forest, alpine meadow, alpine steppe, and alpine desert steppe ecosystems, respectively. Shading denotes the 95% confidence interval of the regression. The boxes denote the 10th and 90th percentiles, and the transverse lines in the boxes denote the average.

**Figure 4** shows the variations of biodiversity–multifunctionality relationships with human pressure in four representative ecosystems across the QTP. There were no obvious linear relationships between biodiversity–multifunctionality correlations and human pressure (**Figure 4**). The biodiversity–multifunctionality correlations in forest and alpine meadow ecosystems underwent a slight downward trend with the enhancement of human pressure (**Figures 4A, C**), whereas the alpine steppe ecosystem experienced a first downward and then upward trend change, but the magnitude of both trends was also small (**Figure 4E**). Compared to the forest and alpine meadow, biodiversity–multifunctionality correlations in alpine desert steppe exhibit an opposite trend change with increased human pressure; similarly (**Figure 4G**), the magnitude of change in this trend is also minor. For the distribution characteristics of biodiversity–multifunctionality correlations in none, low, moderate, and high degrees of human pressure, we found that this relationship in the forest, alpine meadow, and alpine desert steppe ecosystems displayed obvious gradient transformation from none to high levels of human pressure (**Figures 4B, D, H**), with weakening transformation in the forest (average values of correlations in corresponding degrees of human pressure were 0.14, 0.11, 0.09, and 0.08, respectively) and alpine meadow (average values of correlations in corresponding degrees of human pressure were 0.18, 0.16, 0.12, and 0.11, respectively) and enhanced transformation in the alpine desert steppe (average values of correlations in corresponding degrees of human pressure were 0.09, 0.12, 0.14, and 0.15, respectively). For the alpine steppe, although low human pressure possessed the strongest biodiversity–multifunctionality correlations, overall transformation trends decreased from none to high levels of human pressure (the average values of correlations in corresponding degrees of human pressure were 0.17, 0.18, 0.15, and 0.14, respectively; **Figure 4F**).

### 3.3 The effects of climate on the biodiversity–multifunctionality relationships

**Figure 5:**
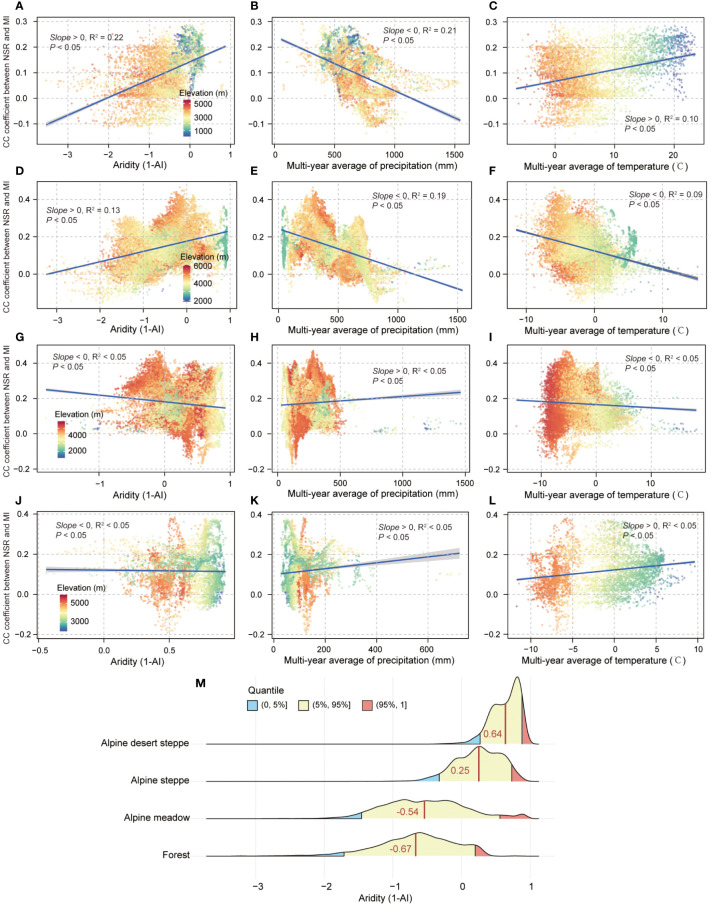
The aridity, precipitation, and temperature in relation to biodiversity–multifunctionality correlations in different ecosystems. **(A**, **D**, **G**, **J)** The bivariate relationships between aridity and biodiversity–multifunctionality correlations in the forest, alpine meadow, alpine steppe, and alpine desert steppe ecosystems, respectively. **(B, E, H, K)** The bivariate relationships between the multiyear average of precipitation and biodiversity–multifunctionality correlations in corresponding ecosystems. **(C, F, I, L)** The bivariate relationships between the multiyear average of temperature and biodiversity–multifunctionality correlations in corresponding ecosystems. Shading denotes the 95% confidence interval of the regression. **(M)** The distribution characteristics of aridity in corresponding ecosystems. Dark red vertical lines denote the average value of aridity (1-AI).

Expanding the analysis for variations of biodiversity–multifunctionality relationships with aridity, precipitation, and temperature found that biodiversity–multifunctionality correlations significantly increased with enhancement of aridity in the forest (average value of 1-AI was −0.67; *Slope* > 0, *R* ^2  ^= 0.22, *p *< 0.05; **Figures 5A, M**) and alpine meadow (average value of 1-AI was −0.54; *Slope* > 0, *R* ^2 ^= 0.13, *p *< 0.05; **Figures 5D, M**). Compared to the first two ecosystems, there were no obvious changes in biodiversity–multifunctionality correlations with increased aridity in the alpine steppe (average value of 1-AI was 0.25; *Slope* < 0, *R* ^2^ < 0.05, *p* < 0.05; **Figures 5G, M**) and alpine desert steppe (average value of 1-AI was 0.64; *Slope* < 0, *R* ^2^ < 0.05, *p *< 0.05; **Figures 5J, M**). For precipitation, we found that the biodiversity–multifunctionality correlations significantly decreased with the increased precipitation in the forest (*Slope *< 0, *R* ^2 ^= 0.21, *p *< 0.05; **Figure 5B**) and alpine meadow ecosystems (*Slope *< 0, *R* ^2 ^= 0.19, *p *< 0.05; **Figure 5E**). By contrast, alpine steppe (*Slope* > 0, *R* ^2^ < 0.05, *p *< 0.05; **Figure 5H**) and alpine desert steppe ecosystems (*Slope* > 0, *R* ^2^ < 0.05, *p *< 0.05; **Figure 5K**) have weaker relationships between biodiversity–multifunctionality correlations and precipitation. For the variations of biodiversity–multifunctionality correlations with temperature, forest ecosystem exhibited a significant positive relationship between them (*Slope* > 0, *R* ^2 ^= 0.10, *p *< 0.05; **Figure 5C**), whereas alpine meadow experienced the opposite trend (*Slope *< 0, *R* ^2 ^= 0.09, *p *< 0.05; **Figure 5F**). Same as precipitation, biodiversity–multifunctionality correlations had no obvious variations with temperature increase in the alpine steppe (*Slope *< 0, *R* ^2^ < 0.05, *p *< 0.05; **Figure 5I**) and alpine desert steppe ecosystems (*Slope* > 0, *R* ^2^ < 0.05, *p *< 0.05; **Figure 5L**).

## 4 Discussion

### 4.1 Spatial patterns of biodiversity–multifunctionality relationships and ecosystem multifunctionality across the QTP

Multiple pieces of evidence have demonstrated that biodiversity is positively correlated with ecosystem multifunctionality, even though the potential mechanisms by which biodiversity is related to ecosystem function remain unclear (Ma et al., 2010; Adler et al., 2011; Jing et al., 2015). Similar results were identified across the QTP; our study confirmed that more than 90% of the region possessed positive biodiversity–multifunctionality relationships (**Figure 3A**), extending previous findings to the grid scale. More importantly, we found that the central region of QTP (mainly involving the Sanjiangyuan region) maintained the highest synergic relationship between biodiversity and ecosystem multifunctionality (**Figure 3A**). This reflects the fact that long-term ecological conservation has a positive effect on improving biodiversity–multifunctionality relationships (Li et al., 2013; Li et al., 2020). Compared with the central region, the eastern region of QTP (mainly involving northwestern Sichuan) has undergone negative biodiversity–multifunctionality relationships (**Figure 3A**). This might be due to the expansion of local industries and land use (such as land degradation as a result of overgrazing in this region) triggered by past economic growth models (Zhou and Peng, 2021), disrupting the relationship between biodiversity and ecosystem multifunctionality. In addition to the relationships between biodiversity and ecosystem multifunctionality across different regional scales (Delgado-Baquerizo et al., 2020; Hu et al., 2021), the geographic variation of environmental context influencing the spatial pattern of ecosystem multifunctionality also needs further attention. Our results indicate that the spatial distribution of ecosystem multifunctionality has undergone a distinct geographical gradient from forest to alpine desert steppe across the QTP (**Figures 2A–C**), as theoretically expected. This finding provides evidence linking the underlying impact of an extensive climatic gradient (with a decreasing trend from forest to alpine desert steppe) across the QTP on the spatial pattern of ecosystem multifunctionality. Since previous works suggest that the geographic variation of climate is one of the main drivers of growth and species distributions for plants along the altitudinal gradient (Sun et al., 2014; Chen J. et al., 2020; Yu et al., 2021), the extent of plant biodiversity will influence the functioning of the ecosystem (Pugnaire et al., 2019); thus, ecosystem multifunctionality is spatially patterned in the same way as climate. Likewise, the climate will also control the matter turnover rates. For example, there are many snow-capped mountains on the alpine steppe and alpine desert steppe of QTP, leading to the turnover efficiency of soil carbon being limited by the persistent low-temperature environment (Ding et al., 2019), thereby determining the low ecosystem multifunctionality principally concentrated in this region. Unlike the alpine steppe and alpine desert steppe, QTP’s forest and alpine meadow were able to maintain a suitable temperature and an adequate water input that will facilitate the normal functioning of ecosystem function (Ban et al., 2022; Li et al., 2022; Lu et al., 2022), ultimately contributing to the maintenance of ecosystem multifunctionality.

### 4.2 The influence of human pressure and climate on biodiversity–multifunctionality relationships

According to the biodiversity-ecosystem functioning concept, species richness and other facets of biodiversity have a significant influence on ecosystem processes (Scherer-Lorenzen et al., 2022). Although numerous studies have been conducted on the interaction between biodiversity and ecosystem functioning (Soliveres et al., 2014; Jing et al., 2015; Meyer et al., 2018; Delgado-Baquerizo et al., 2020; Hu et al., 2021), these studies have largely focused on the identification of the biodiversity–ecosystem relationship, ignoring the probable disturbance of human pressure on biodiversity–multifunctionality relationships in the alpine region. Fortunately, our study showed that the influence of human pressures on biodiversity–multifunctionality relationships was nonsignificant in all ecosystems of QTP (**Figure 4**). This might be due to policy implementation to control ecological degradation and enhance human well-being (Chen et al., 2014; Pan et al., 2016; Pan et al., 2017; Wang and Wei, 2022), such as the Regional Ecological Construction and Environmental Protection Plan on the QTP, Tibet Ecological Protection Barrier Protection and Construction Plan, and Overall Plan for Ecological Protection and Construction of Qinghai Sanjiangyuan Nature Reserve (Wang and Wei, 2022), leading to human activities being mainly concentrated in urban and scenic areas and the discontinuous variation of human pressures across the QTP (Wei et al., 2022), thereby offsetting the negative influence of human pressures on biodiversity–multifunctionality relationships on the QTP (**Figures 4B, D, F**) and ultimately generating the ambiguous pattern between human pressure and biodiversity–multifunctionality relationships (**Figure 4**). The variation of biodiversity–multifunctionality relationships with different degrees of human pressure in alpine desert steppe ecosystem further supplements the uncertain pattern. We found the synchronicity between biodiversity–multifunctionality correlations and enhancement of human pressure (**Figures 4G, H**), indicating that positive human activities (such as strengthening of management to combat land degradation and desertification) may improve the background conditions in desert areas and enhance synergic relationships between biodiversity and multifunctionality (Liu et al., 2021; You et al., 2021).

In contrast to human pressure, aridity significantly strengthened the synergic relationship between biodiversity and ecosystem multifunctionality in the forest and alpine meadow ecosystems (**Figures 5A, D**). In the forest, the biodiversity–multifunctionality relationship was significantly negatively and positively related to precipitation (**Figure 5B**) and temperature (**Figure 5C**), respectively. It is possible that there is a steeper decline in ecosystem function, such as soil microorganism activity weakening with increasing soil moisture in forests that receive ample precipitation all year round (Schafer and Kotanen, 2003; Zhang and Zhang, 2016; Chen Y. et al., 2020), possibly leading to a large amount of organic matter not being decomposed by soil microorganisms due to respiration limitations (Medrano et al., 2015; Trugman et al., 2019), thereby exhibiting this consequence of negative correlation between biodiversity–multifunctionality relationship and precipitation (**Figure 5B**). This can be explained by the fact that biodiversity–multifunctionality correlations gradually increased with temperature (**Figure 5C**), because warming can both evaporate excess water and enhance the decomposition capacity of soil microorganisms to produce more compounds that can be more easily absorbed by vegetation (Chou and Lan, 2012; Levy et al., 2019; Sun et al., 2020). The positive association of aridity with geographic variation of biodiversity–multifunctionality relationship (**Figure 5A**) also further contributes to answering the significant question that excess water (**Figure 5M**) is adverse to ecosystem function performance (such as mineralization and nitrification of nitrogen) and biodiversity maintenance in the forest (Zhao and Li, 2017; Pugnaire et al., 2019; Sun et al., 2020; Ye et al., 2020; Sun et al., 2022). Indeed, the strong positive correlation between the biodiversity–multifunctionality relationship and aridity (**Figure 5D**) in the alpine meadow is also possibly attributed to the obstacle of excess water (**Figures 5E, M**). For example, there are some alpine meadow areas that are always under waterlogged conditions, which could lower the soil’s oxygen content and increase the mortality of soil microorganisms (Wang X. et al., 2021; Sun et al., 2022), decreasing the potential for ecosystem function complementarity and, as a result, weakening the biodiversity–multifunctionality relationship in the more humid regions (**Figures 5D, M**).

### 4.3 Uncertainties and future directions

Our study underscored the obvious fact that aridity significantly affects the geographic variation of biodiversity–multifunctionality relationships across the forest and alpine meadow ecosystems of QTP. In contrast to links between human pressure or climate and biodiversity–multifunctionality relationships in this study, actual interactions between matter, energy, and organisms are dependent on the temporal and spatial dynamics of the change process in species composition and anthropogenic and natural disturbances across coupled ecosystems (Ratcliffe et al., 2017; Gounand et al., 2018; Ehbrecht et al., 2021; Scherer-Lorenzen et al., 2022), which are not reflected in our spatial analysis of functional links between them. Acknowledging this, future studies should consider potential mechanisms of exchange between matter, energy, and organisms at a plot level because our comprehensive understanding of how different ecosystems respond to changes in resource availability can be greatly improved by reflecting the ecological process in this way (Kulmatiski et al., 2008; Pugnaire et al., 2019). While our findings support much of the mechanistic and experimental research related to the spatial link between ecosystem multifunctionality and biodiversity (Berdugo et al., 2017; Ehbrecht et al., 2021; Ma et al., 2021; Moi et al., 2022), we must emphasize that cross-correlations are not necessarily a sign of causality. In addition, the currently ongoing global pattern of vascular plant diversity provides a preliminary assessment of the potential level of plant diversity. Future work is therefore required to trigger the development of new methodologies and metrics to assess these proxies of plant and soil biodiversity on our planet. Finally, more meteorological sites are not available; therefore, future study should take into account relating the actual to high-precision satellite products.

## 5 Conclusions

By reflecting extensive changes in the geographic gradient of ecosystem multifunctionality with climatic variation across different ecosystems based on multivariate datasets, we report results that show the spatial distribution of biodiversity–multifunctionality relationships at the alpine scale can inform our comprehensive understanding of species distribution and dynamics. Our results suggest that biodiversity and multifunctionality are synergistically related in most areas of the QTP. We have also shown that human pressure has a negative impact on biodiversity–multifunctionality relationships in the forest, alpine meadow, and alpine steppe ecosystems, but with large contrasts in the alpine desert steppe ecosystem, which contributes to the important implications for desertification control that need to be further implemented in the QTP. An important finding of our study is that there is a significant positive association between aridity and biodiversity–multifunctionality relationships in forest and alpine meadow ecosystems. These results demonstrate that climate has a more significant impact on biodiversity–multifunctionality relationships relative to human pressure and the urgency of targeted management actions to monitor the influence of climate change on biodiversity and ecosystem functions across the QTP, especially in forest and alpine meadow ecosystems.

## Data availability statement

The original contributions presented in the study are included in the article/supplementary material. Further inquiries can be directed to the corresponding author.

## Author contributions

CY: Conceptualization (lead); formal analysis (lead); methodology (lead); visualization (lead); writing – original draft (lead); writing – review and editing (lead). SW: Funding acquisition (lead); Methodology (supporting); visualization (supporting); writing–review and editing (supporting). YW: Methodology (supporting); validation (supporting); writing–review and editing (supporting). TZ: Methodology (supporting); validation (supporting); writing–review and editing (supporting). RL: Methodology (supporting). All authors contributed to the article and approved the submitted version.
